# Small and Large Extracellular Vesicles of Porcine Seminal Plasma Differ in Lipid Profile

**DOI:** 10.3390/ijms25137492

**Published:** 2024-07-08

**Authors:** Pablo Martínez-Díaz, Ana Parra, Christian M. Sanchez-López, Josefina Casas, Xiomara Lucas, Antonio Marcilla, Jordi Roca, Isabel Barranco

**Affiliations:** 1 Department of Medicine and Animal Surgery, Faculty of Veterinary Science, University of Murcia, 30100 Murcia, Spain; pablo.martinezd@um.es (P.M.-D.); ana.parra@um.es (A.P.); xiolucas@um.es (X.L.); isabel.barranco@um.es (I.B.); 2 Àrea de Parasitologia, Departament de Farmàcia i Tecnologia Farmacèutica i Parasitologia, Universitat de València, 46100 Valencia, Spain; christian.sanchez@uv.es (C.M.S.-L.); antonio.marcilla@uv.es (A.M.); 3 Joint Research Unit on Endocrinology, Nutrition and Clinical Dietetics, Health Research Institute La Fe, Universitat de València, 46100 Valencia, Spain; 4 Research Unit on BioActive Molecules (RUBAM), Institute for Advanced Chemistry (IQAC-CSIC), Jordi Girona 18-26, 08034 Barcelona, Spain; fina.casas@iqac.csic.es; 5 Centro de Investigación Biomédica en Red de Enfermedades Hepáticas y Digestivas (CIBEREHD), Instituto de Salud Carlos III, 28029 Madrid, Spain

**Keywords:** extracellular vesicles, lipidomics, porcine, seminal plasma, subsets

## Abstract

Seminal plasma contains a heterogeneous population of extracellular vesicles (sEVs) that remains poorly characterized. This study aimed to characterize the lipidomic profile of two subsets of differently sized sEVs, small (S-) and large (L-), isolated from porcine seminal plasma by size-exclusion chromatography and characterized by an orthogonal approach. High-performance liquid chromatography–high-resolution mass spectrometry was used for lipidomic analysis. A total of 157 lipid species from 14 lipid classes of 4 major categories (sphingolipids, glycerophospholipids, glycerolipids, and sterols) were identified. Qualitative differences were limited to two cholesteryl ester species present only in S-sEVs. L-sEVs had higher levels of all quantified lipid classes due to their larger membrane surface area. The distribution pattern was different, especially for sphingomyelins (more in S-sEVs) and ceramides (more in L-sEVs). In conclusion, this study reveals differences in the lipidomic profile of two subsets of porcine sEVs, suggesting that they differ in biogenesis and functionality.

## 1. Introduction

Seminal plasma is the complex biofluid that surrounds spermatozoa during and after ejaculation. It is composed of a mixture of the secretions from the functional organs of the male reproductive tract, mainly from the epididymis and the accessory sex glands [1]. Seminal plasma contains a wide variety of molecules that play key roles in crucial reproductive processes, such as the modulation of sperm functions [2,3] and the induction of maternal immune tolerance to spermatozoa and embryos [4,5]. Some of these biomolecules, including proteins [6], lipids [7], small non-coding and regulatory RNAs [8], are not only circulate freely in seminal plasma, but are also loaded into cell-derived membrane-surrounded nanovesicles, called extracellular vesicles (EVs), where biomolecules are protected from the degradative activity of seminal plasma enzymes [9,10].

Extracellular vesicles play a key role in cell-to-cell communication and circulate freely in all body fluids [11]. Seminal EVs (sEVs), like those of any other body fluid, are a heterogeneous population with different phenotypic characteristics [6,12,13]. This heterogeneity suggests the existence of different sEV subsets, which may have different cell origin, biogenesis pathways, cargoes, target cells and functional performance [14]. The isolation and separate characterization of EV subtypes circulating in body fluid remains a scientific challenge [15].

Two subsets of sEVs of different sizes, termed small sEVs (S-sEVs) and large sEVs (L-sEVs), were successfully isolated from porcine seminal plasma using a size-exclusion chromatography (SEC)-based isolation procedure [16]. Such size-associated EV subsets are in line with the recommendation of the International Society for Extracellular Vesicles (ISEV) to use terms referring to phenotypic characteristics, such as size, to define EV subsets [17]. The proteomic profile of these two sEV subsets revealed quantitative differences between S-sEVs and L-sEVs in several proteins [6]. Such differences in protein cargo support the concept of the coexistence of different sEV subsets in porcine seminal plasma, which may have differences in their functionality, as EV functions are closely related to their cargo [14,18].

In addition to proteins, EVs also contain lipids, a diverse family of macromolecules that perform essential biological functions, including structural ones, as they are essential components of membranes, energy storage and signaling [19]. The lipid composition of EVs is determined by the biogenesis pathway in the cell of origin and contributes to their intracellular trafficking, as well as their uptake and functional response in recipient cells [18,20]. The lipid content of sEVs has received much less attention than that of proteins or RNAs [10], and quantitative lipidomic studies of EVs are needed [21]. Quantitative studies of the lipidomic profile of sEVs are essential for a better understanding of the underlying mechanisms of EV biogenesis and protein packaging, as well as for the exploration of sources of molecular biomarkers of fertility in sEVs [22].

The aim of the present study was to provide a detailed lipidomic profile of two differently sized porcine sEV subsets using high-performance liquid chromatography (HPLC) coupled to high-resolution mass spectrometry (HRMS), a high-throughput and highly sensitive technology that allows comprehensive and quantitative analysis of a broad repertoire of lipid species in any biological sample, including EVs [23]. To the best our knowledge, there are only four studies that have reported the relative amounts of some lipid classes in sEVs, two in humans [24,25], one in horses [26], and one in pigs [7]. Only the study by Brouwers et al. was performed using MS-based technology for relative lipid quantification [25], the other three studies used methods based on thin-layer chromatography (TLC), an older technology that is less sensitive than MS-based technology [27].

## 2. Results

### 2.1. Characterization of sEVs

**Figure 1:**
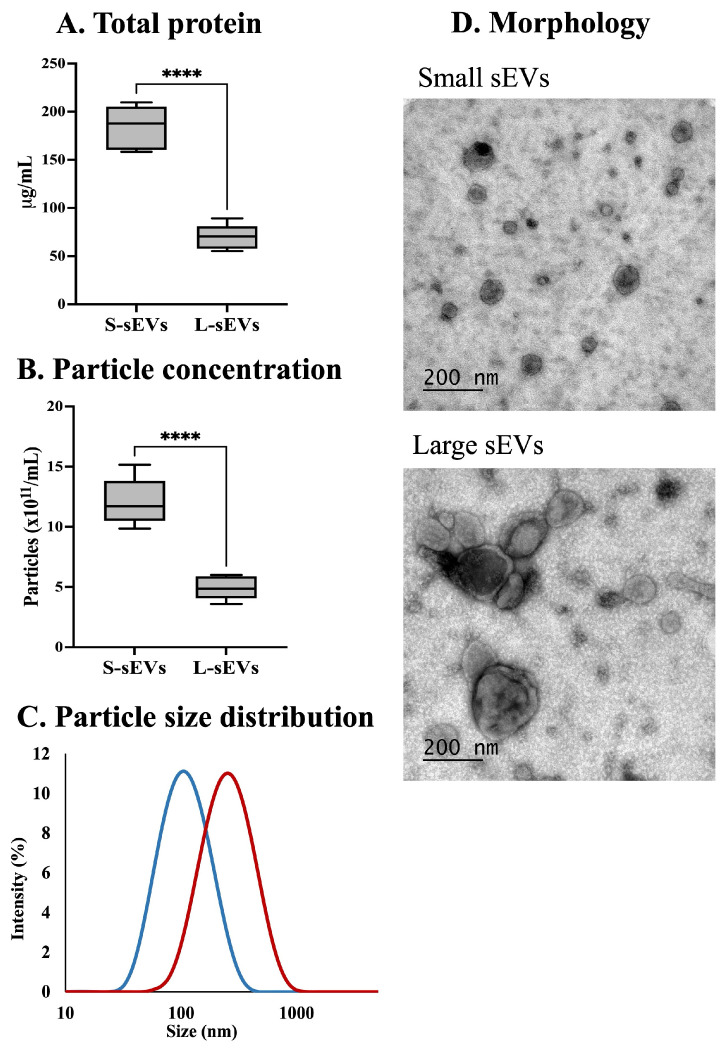
Phenotypic characterization of large (L) and small (S) porcine seminal extracellular vesicles (sEVs). Box plots showing (**A**) total protein concentration and (**B**) particle concentration measured by nanoparticle tracking analysis. (**C**) Particle size distribution measured by dynamic light scattering analysis (blue line indicates S-sEVs and red line indicates L-sEVs). (**D**) Representative transmission electron microscopy images showing the morphology of sEVs. **** *p* < 0.0001. Box plots: Boxes enclose the 25th and 75th percentiles, whiskers extend to the 5th and 95th percentiles, and line represents median.

Total protein concentration was higher (*p* < 0.0001) in the S-sEV samples (mean ± SD: 184.76 ± 21.24 μg/mL) than in the L-sEV samples (70.35 ± 13.22 μg/mL) (Figure 1A). The nanoparticle tracking analysis (NTA) showed that the particle concentration (mean ± SD) was higher (*p* < 0.0001) in S-sEV samples (12.07 × 10^11^ ± 1.92 × 10^11^ particles/mL) than in L-sEV samples (4.89 × 10^11^ ± 0.95 × 10^11^ particles/mL) (Figure 1B). It is interesting to note that the number of particles per μg of protein in the S-sEVs samples (6536.45 ± 695.50 particles/μg protein) was similar to that in the L-sEVs samples (7358.61 ± 2687.63 particles/μg protein). The dynamic light scattering (DLS) analysis showed that the particles were smaller (*p* < 0.01) in the S-sEV samples (119.63 ± 17.46, 78 and 155 nm for the 25th and 75th percentiles) than in the L-sEV samples (282.51 ± 26.61, 160 and 330 nm for the 25th and 75th percentiles) (Figure 1C). The transmission electron microscopy (TEM) images confirmed that the S-sEV samples contained smaller EVs than the L-sEV samples, and further showed that the EVs in the S-sEV samples were mostly spherical in shape, whereas those in the L-sEV samples were more heterogeneous in shape (Figure 1D).

**Figure 2:**
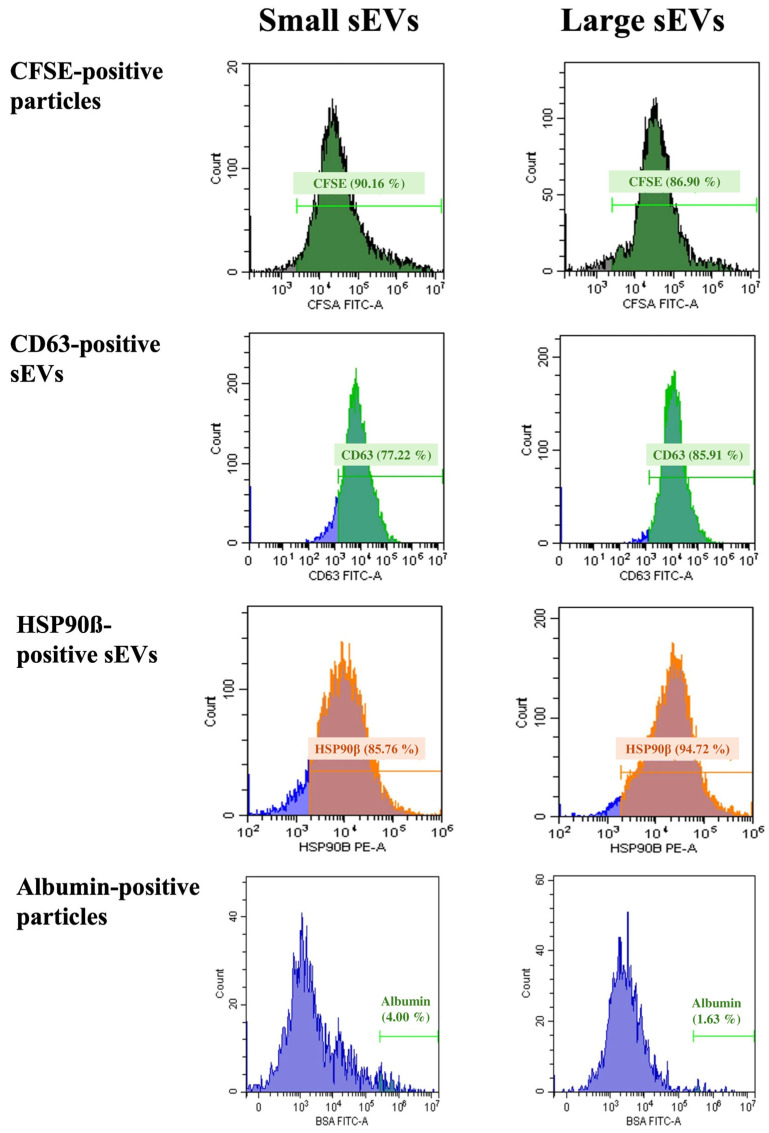
Representative flow cytometry plots (violet side scatter [violet-SSC]/direct side scatter [FSC]) showing CFSE, CD63, HSP90β and albumin positive events in samples of small and large seminal extracellular vesicles (sEVs) isolated from porcine seminal plasma.

Flow cytometric analysis showed that most of the particles analyzed were EVs, as they were positive for carboxyfluorescein succinimidyl ester (CFSE). The percentages (mean ± SD) were similar for S-sEVs (79.64% ± 3.83%) and L-sEVs (80.89% ± 5.15%). Flow cytometry analysis also revealed high and similar levels of CD63 and HSP90β expression in S-sEV and L-sEV samples (Figure 2). Specifically, the percentages of S-sEVs and L-sEVs expressing CD63 were 79.62% ± 1.10% and 81.85% ± 4.54%, respectively. The percentages of S-sEVs and L-sEVs expressing HSP90β were 80.75% ± 5.98% and 88.06% ± 2.66%, respectively. Finally, flow cytometry analysis showed that samples of S-sEVs and L-sEVs had low albumin content. Specifically, the percentage of albumin-positive particles was 3.84% ± 1.47% in S-sEVs samples and 2.28% ± 0.75% in L-sEVs samples (Figure 2).

### 2.2. Lipids Identified in sEVs by LC–MS Lipidomic Analysis

**Figure 3:**
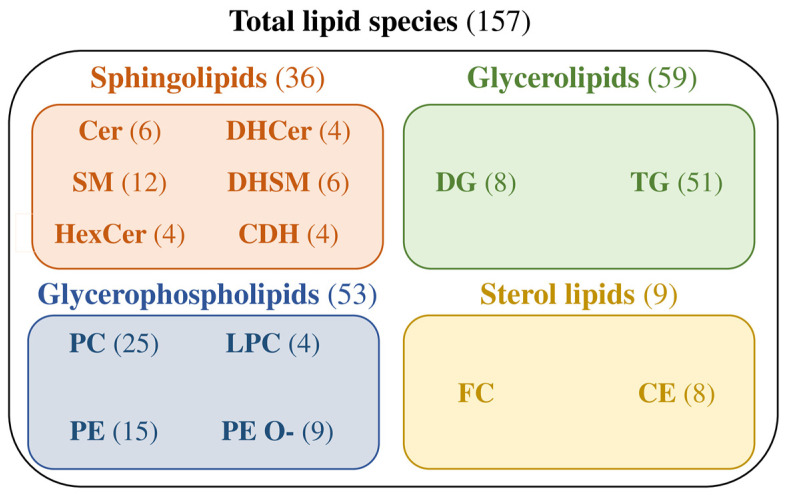
Lipid species identified and quantified in porcine seminal extracellular vesicles distributed by lipid categories (each with a different box color) and lipid classes within each lipid category. The data show the number of lipid species that were identified and quantified. Cer: ceramides; DHCer: dihydroceramides; SM: sphingomyelin; DHSM: dihydrosphingomyelin; HexCer: hexosylceramides; CDH: ceramide dihexoside; PC: phosphatidylcholines; LPC: lyso-phosphatidylcholines; PE: phosphatidylethanolamines; PE O-: ether-linked phosphatidylethanolamines; DG: diacylglycerols; TG: triacylglycerols; FC: free cholesterol; CE: cholesteryl esters.

The targeted lipidomic analysis identified and quantified a total of 157 lipid species belonging to four major lipid categories, namely sphingolipids (SP), glycerophospholipids (GP), glycerolipids (GL), and sterol lipids (ST). The identified and quantified lipid species belong to 14 lipid classes, specifically six SP, four GP, two GL and two ST classes (Figure 3).

The 36 SP species identified and quantified were six ceramides (Cer), four dihydroceramides (DHCer), 12 sphingomyelins (SM), six dihydrosphingomyelins (DHSM), four hexosylceramides (HexCer), and four ceramide dihexosides (CDH). The 53 GP species were 25 phosphatidylcholines (PC), 15 phosphatidylethanolamines (PE), four lysophosphatidylcholines (LPC), and nine ether-linked PE (PE O-). The 59 GL species were eight diacylglycerols (DG) and 51 of triacylglycerols (TG). Finally, the nine ST species were free cholesterol (FC) and eight cholesteryl esters (CE) (Figure 3 and Table S1). Lipid species identified and quantified in both sEV subsets were uploaded to the Vesiclepedia database (Study ID: 3594) [28].

### 2.3. Comparison of Lipidomic Profile between S-sEVs and L-sEVs

**Figure 4:**
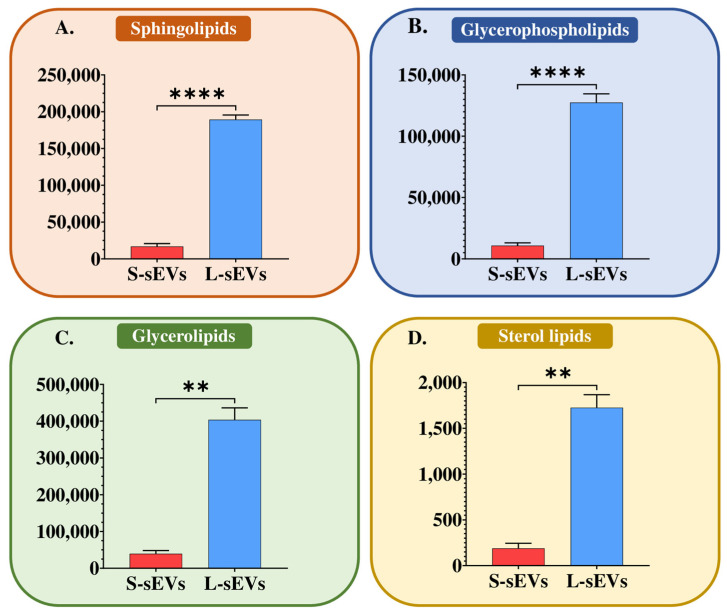
Histograms showing the differences between large (L-) and small (S-) seminal extracellular vesicles (sEVs) in the relative abundance of sphingolipids (**A**), glycerophospholipids (**B**), glycerolipids (**C**), and sterol lipids (**D**). Data are expressed as pmol eq/mg protein and are the mean ± SD. **** *p* value < 0.0001, ** *p* value < 0.01.

**Figure 5:**
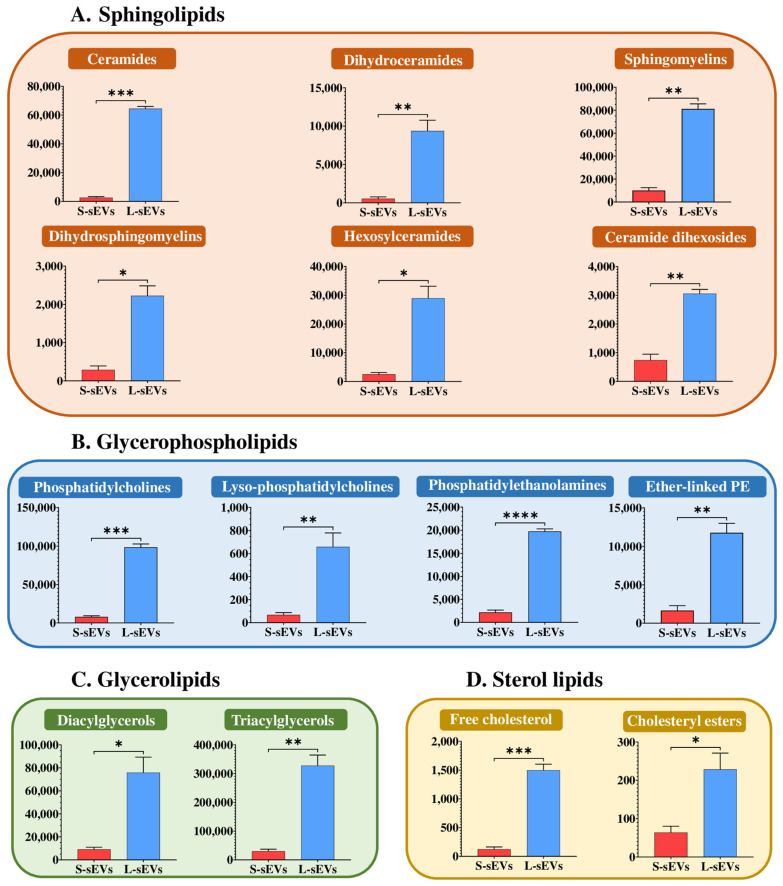
Histograms showing the differences between large (L-) and small (S-) porcine seminal extracellular vesicles (sEVs) in the relative abundance of identified and quantified lipid classes distributed by lipid categories: sphingolipids (**A**), glycerophospholipids (**B**), glycerolipids (**C**), and sterol lipids (**D**). Data are expressed as pmol eq/mg protein and are the mean ± SD. **** *p* value < 0.0001, *** *p* value < 0.001, ** *p* value < 0.01, * *p* value < 0.05.

Qualitative differences between S-sEVs and L-sEVs were found only in two CE species. Specifically, CE (16:0) and CE (20:2) were identified and quantified only in the S-sEVs. They accounted for 1.3% of the total lipid species that were identified and quantified in the S-sEV samples. In contrast, quantitative differences were found in virtually all identified and quantified lipid classes. The lipid to protein ratio of the two subsets of sEVs was different, being 5:1 for S-sEVs and 10:1 for L-sEVs. L-sEVs had higher relative amounts (*p* < 0.01) of the four major lipid categories, namely SP, GP, GL, and ST, compared to S-sEVs (Figure 4A–D). All the 14 identified and quantified lipid classes were more abundant in L-sEVs than in S-sEVs (*p* < 0.05), regardless of their lipid category (Figure 5A–D).

**Figure 6:**
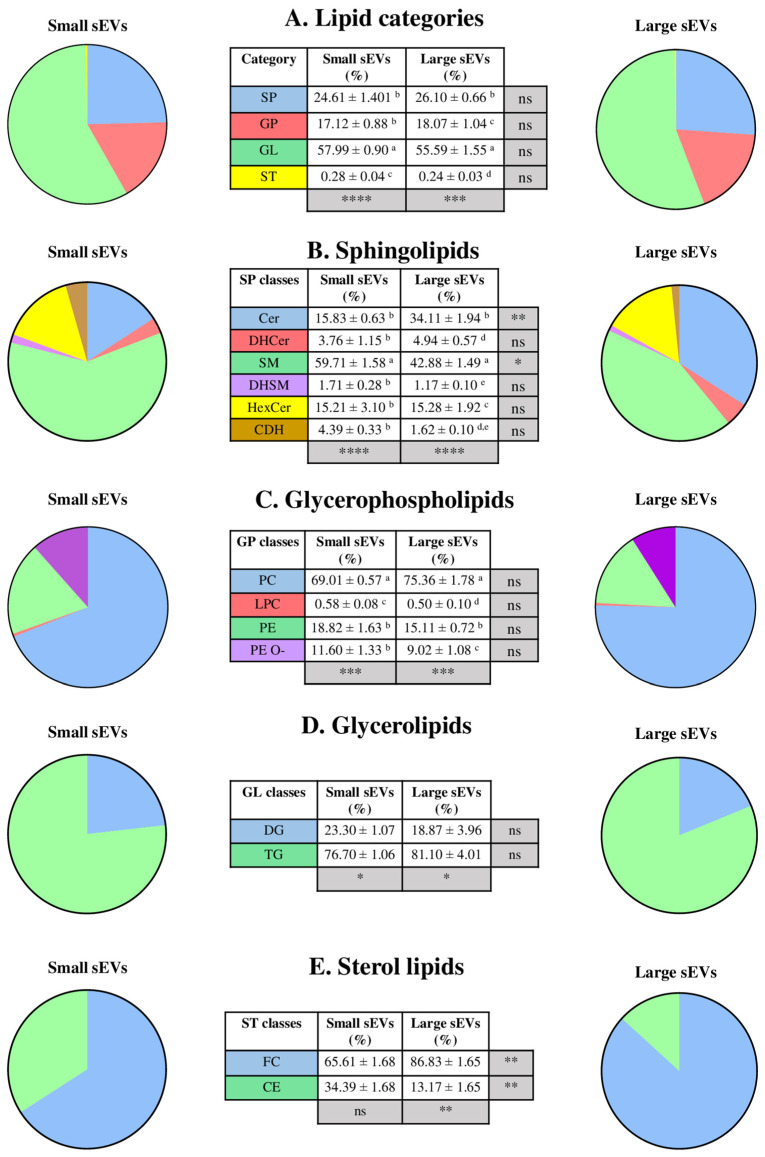
Pie charts and supplementary tables showing the distribution of different categories (**A**) and classes of lipids (**B**–**E**) between small (S-) and large (L-) porcine seminal extracellular vesicles (sEVs). Data in tables show the mean percentage (%) ± SD. **** *p* value < 0.0001, *** *p* value < 0.001, ** *p* value < 0.01, * *p* value < 0.05, ns *p* value > 0.05. a, b, c, d, e indicates differences at *p* < 0.05 between lipid classes within a lipid category. SP: sphingolipids; GP: glycerophospholipids; GL: glycerolipids; ST: sterol lipids; Cer: ceramides; DHCer: dihydroceramides; SM: sphingomyelin; DHSM: dihydrosphingomyelin; HexCer: hexosylceramide; CDH: ceramide dihexoside; PC: phosphatidylcholine; LPC: lyso-phosphatidylcholine; PE: phosphatidylethanolamine; PE O-: ether-linked phosphatidylethanolamine; DG: diacylglycerols; TG: triacylglycerols; FC: free cholesterol; CE: cholesteryl esters.

The four lipid categories were similarly distributed in S-sEVs and L-sEVs. GL was the dominant category with more than 50%, while ST was the minority with less than 1% (Figure 6A). Regarding the lipid classes within each lipid category, in the SP category, SM, Cer and HexCer were the most predominant lipid classes in both sEVs subsets. However, SM and Cer were present in different proportions in S-sEVs and L-sEVs (*p* < 0.05). SM were proportionally more abundant in S-sEVs than in L-sEVs, whereas Cer were proportionally more abundant in L-sEVs than in S-sEVs (Figure 6B). In the GP category, PC and PE were the predominant lipid classes in both sEV subsets, with no differences between sEV subsets (Figure 6C). In the GL category, TG was the more predominant lipid class in both sEV subsets, and the distribution of TG and DG was similar in L-sEVs and in S-sEVs (Figure 6D). In the ST category, FC was the predominant lipid in both sEV subsets, but FC and CE were present in different proportions in S-sEVs and L-sEVs (*p* < 0.01). FC was proportionally more abundant in L-sEVs than in S-sEVs, whereas CE were proportionally more abundant in S-sEVs than in L-sEVs (Figure 6E).

## 3. Discussion

In the present study, the lipidomic profiles of two subsets of EVs of different sizes isolated by SEC from porcine seminal plasma (sEVs), termed small sEVs (S-) and large sEVs (L-), were characterized. Lipidomic studies of EVs are still limited, and those characterizing the lipidomic profiles of EV subpopulations are even more limited. To the best of our knowledge, only a few studies have compared the lipid profile of EV populations of different sizes, all of which are secreted in vitro from body cells or single cell lines [29,30,31,32]. Therefore, the present study is the first to compare the lipidomic profile of EV subsets of different sizes circulating in body fluids. The results of the above-mentioned studies showing differences between small and large EVs in the relative amounts of several lipid species are consistent with those reported here in that the relative amounts of many lipid classes differ between S-sEVs and L-sEVs. 

Separation of the two sEV subsets was achieved by centrifugation at 20,000× *g*, a g-force that allows the sedimentation of the large particles, including the L-sEVs, while leaving the smaller particles, including S-sEVs, in the supernatant [33]. Both subsets of sEVs were then separately purified by SEC, a procedure that allows the recovery of a large number of intact and minimally contaminated EVs [15]. The samples of sEVs were characterized using an orthogonal approach and the results confirmed that S-sEVs and L-sEVs differed in size, as expected, but also in concentration and morphology, in agreement with previous studies [6,34]. Flow cytometry analysis showed that the isolated sEVs in both S-sEV and L-sEV samples exhibited a high degree of purity, a necessary requirement to obtain reliable results in EV composition studies such as the present one [21]. It should be noted that the major non-EV particles co-isolated in EV samples are lipoproteins, which are characterized by a hydrophobic lipid core [35]. Lipoproteins are usually co-isolated with EVs due to their similar size to smaller EVs (≈25–80 nm for lipoproteins). This can lead to the overestimation of some lipid species [21,36,37].

Extracellular vesicles are membranous structures derived from cell membranes. Therefore, the lipid repertoire of EVs is expected to consist mainly of cell membrane lipids. However, they may contain small amounts of other cytosol-derived lipids acquired during biogenesis [11,21]. Indeed, the most abundant lipids found in both sEV subtypes were, in descending order, TG, PC, SM, DG, and Cer. These results would be consistent with those reported by Peterka et al., who found that TG, PC and SM were some of the most abundant lipids in EVs isolated from human plasma [38]. Most of the lipids found in the sEVs are typical structural lipids of cell membranes. In particular, PC, SM and Cer are abundant structural lipids in plasma and endosomal membranes [39]. In contrast, TG and DG are lipid classes present in the inner space of the bilayer but are not thought to play a structural role [38]. This is the first study to report the presence of TG and DG in sEVs, lipid classes that have also been found in EVs isolated from human blood plasma [38], mouse urine [40], and human brain tissue [41]. The enrichment of TG in both sEV subsets should be viewed with caution, as TG are important components of lipoproteins and lipid droplets [42], which are common contaminants of EV samples, as discussed above. However, it is unlikely that the high levels of TG found in porcine sEV samples are solely due to lipoprotein contamination. This contention would be supported by flow cytometry results showing a very high percentage of CFSE+ events in both sEV samples, which would be membrane-intact sEVs [43]; and by the relatively low amount of CE, another lipid class abundant in lipoproteins [21,44]. Furthermore, TG have been found at high levels in EVs produced in vitro by cell lines [45], which are less contaminated by lipoproteins than those isolated from body fluids [21]. The high levels of TG found in sEVs may be due to the binding of free lipoproteins circulating in seminal plasma to sEV membrane, which has been shown to occur in EVs cultured in vitro [37,46]. It should be noted here that the comparison of the lipidomic profile of EVs isolated from different body fluids or different cell cultures may not be scientifically accurate. The lipid composition of EV samples is strongly influenced by the isolation procedure used [47], in addition to the expected natural influencing factors such as the biogenesis pathway, the cell of origin and its functional state, i.e., physiological or pathological [11]. Among other reasons, the variety of methods used for EV isolation leads to the unpredictable presence of non-EV particles in isolated EV samples, including lipoproteins as mentioned above. This can lead to inaccurate results regarding the lipid composition of EVs, which makes comparisons between studies of little scientific value.

In addition to the structural role of lipids in EVs, their role in cell-to-cell communication must also be considered. Lipids are one of the active molecules that facilitate the transport EVs from source cells to target cells [11,47]. With respect to sEVs, it is well known that they bind to and deliver their cargo to spermatozoa, thereby regulating important sperm functions such as capacitation [10]. Leahy et al. showed enrichment of the sperm proteome after sEV binding, which would indicate transfer of proteins from sEVs to spermatozoa [48]. Lipid transfer from sEVs to sperm remains to be demonstrated [49], although the easy transfer of lipophilic dye between epididymosomes and spermatozoa [50] suggests that lipid exchange occurs between sEVs and spermatozoa.

Lipids are known to be involved in key sperm functions such as capacitation. Certainly, key steps in sperm capacitation are the removal of cholesterol from the sperm surface and the subsequent remodeling of membrane lipid to increase membrane lipid fluidity [51,52]. Sperm subjected to stressful conditions, such as cryopreservation, undergo so-called premature or false capacitation, rendering them incapable of fertilization [53]. Binding of sEVs to spermatozoa prevents premature or false capacitation and stabilizes sperm membranes, including acrosomal membranes [54,55,56]. This role of sEVs may be mediated, at least in part, by the transfer of certain lipid species from sEVs to spermatozoa. Indeed, some of the lipids found in the sEVs are associated with processes related to sperm physiology. For instance, some specific PC and PE species that were detected in both sEV subsets, namely PC (36:1), PC (38:4), and PE (34:4), have been proposed as sperm motility markers [57]. In addition, PC in particular, but also PE, is known to play a key role in the prevention of phospholipid movement by binding to seminal plasma proteins, thereby maintaining sperm membrane stability [58]. It has also been shown that the stabilizing effect of PC on the sperm membrane prevents cold shock-induced ultrastructural damage in porcine spermatozoa [59]. The degradation of PC by phospholipase A2 results in LPC, which has been measured in S-sEV and L-sEV samples and could be another sEV lipids that modulates sperm functionality, specifically the acrosomal reaction, as moderate amounts of LPC have been shown to induce the acrosomal reaction in bovine and human spermatozoa [60,61,62]. PC can also be degraded to DG, which are abundant in the sperm membrane and whose reduction is associated with infertility in bulls [63]. Concentrations of TG and FC in seminal plasma have been shown to be significantly higher in cats with good than poor semen quality [64]. Loss of sperm FC is a key early step in capacitation, as noted above. SM plays an important role in slowing down FC loss, thereby influencing the timing of sperm capacitation [65]. Therefore, it is plausible that one of the ways by which sEVs avoid premature capacitation is through the transfer of SM to spermatozoa. L-sEVs would be more effective in modulating sperm function via these lipid-dependent pathways because they contain higher levels of all these lipid classes than S-sEVs. Recent evidence has shown that sEVs decrease lyso-PC in porcine spermatozoa [66]. Thus, sEVs may also be involved in the removal of “unneeded” molecules from spermatozoa, as suggested by Leahy et al. [48].

Lipidomic analysis revealed qualitative differences in two CE species, specifically CE (16:0) and CE (20:2) between the two sEV subsets, which were identified in S-sEVs but not in L-sEVs. CEs are energy storage lipids formed by the esterification of cholesterol to fatty acids [67] and, as mentioned above, are abundant in the hydrophobic core of lipoproteins. Therefore, the interaction of S-sEVs with lipoproteins circulating in the seminal plasma may be responsible for the presence of these two CEs in S-sEVs. Small EVs are more likely than large EVs to be surrounded by a peripheral coronal layer composed of active molecules, including lipoproteins [68,69,70]. These fatty acid-bearing cholesterol lipids may indicate the pool of fatty acids available to synthetize complex lipids [71]. One of the two CEs present only in S-sEVs was CE (16:0), termed cholesteryl palmitate, which is formed by the esterification of cholesterol and palmitic acid (C16:0). Recently, Barreca et al. developed an innovative approach for the metabolic labeling of a homogeneous population of small EVs based on the tracking of a fluorescent palmitic acid analogue, called Bodipy FL C16 [72]. They successfully demonstrated the localization of BODIPY FL C16 in specific intracellular compartments, including the endoplasmic reticulum and the endolysosomal compartment, while it was absent from the cell membrane of EV-producing cells. Therefore, they hypothesized an association between palmitic acid and the intracellular origin of a specific subpopulation of small EVs. The exclusive presence of CE (16:0) in S-sEV samples would support that these samples are enriched with exosomes (i.e., endosome-derived EVs) whose size matches that of S-sEVs. The other uniquely identified CE in S-sEVs, CE (20:2), has not been previously reported. Whether these two lipids play a relevant role in S-sEVs is therefore unknown. In contrast to the few qualitative differences, there were clear quantitative differences in the lipid profile of S- and L-sEVs. L-sEVs had higher relative amounts of the four major lipid categories and of each of the 14 identified lipid classes. Since the number of sEVs/μg protein was similar in both sEV subsets, size differences between S- and L-sEVs could be the reason for these quantitative differences. In this and previous studies [21], it has been shown that the lipidomic profile of EVs is largely composed of membrane lipids. Therefore, it is reasonable to assume that larger EVs have a greater relative amount of lipids than smaller EVs due to their larger membrane surface area.

In addition to the relative amount, the proportion of each lipid category and class was also compared between S-sEVs and L-sEVs. The four lipid categories were similarly distributed in S-sEVs and L-sEVs, which would be consistent with the results reported by Durcin et al. and Kim et al. in subtypes of EVs of different sizes from adipocytes and a specific cell line (DU145 HRPC), respectively [29,31]. In terms of lipid classes, SM and Cer were observed to have the most notable proportional differences between the two subsets of sEVs. Hydrolysis of SM to Cer is a known pathway in the biogenesis of both exosomes and ectosomes [11]. It is plausible that this pathway is more active in L-sEVs than in S-sEVs, because L-sEVs had a higher Cer:SM ratio than S-sEVs. It is interesting to note that L-sEVs contain more ectosomes than exosomes [73].

The quantitative lipidomic differences between S- and L-sEVs suggest differences in functional roles between the two sEV-subsets. Indeed, a recent functional study in pigs by Barranco et al. showed that L-sEVs were able to modulate sperm metabolism, whereas S-sEVs had no detectable effect [74]. The different molecular composition of S- and L-sEVs may explain the observed functional differences between the two subsets of sEVs. This would be supported by the results of Barranco et al. who reported quantitative differences between the two subsets of sEVs in proteins related to sperm functionality [6]. Unfortunately, lipidomics does not have the same background of functional data as proteomics. Therefore, investigating the role of sEV lipids in modulating sperm function remains a challenge.

## 4. Materials and Methods

### 4.1. Animals and Seminal Plasma Samples

Animal procedures were performed in accordance with international guidelines for the protection of animals used for research purposes (Directive 2010-63-EU) and the experiment carried out was approved by the Bioethics Committee of the University of Murcia at its meeting on 25 March 2021 (research code: CBE: 367/2020). The reagents used were provided by Merck (Darmstadt, Germany) unless otherwise stated.

Nine ejaculates were collected from nine mature, healthy, and fertile Pietrain boars (one ejaculate per boar) housed in an artificial insemination (AI) center of AIM Iberica (Topigs Norsvin España SLU). The boars were routinely used to produce AI semen doses for commercial AI programs, and the ejaculates used in this study met the quality criteria established for the commercial production of AI semen doses, namely greater than 200 × 10^6^ sperm/mL with more than 70% motile spermatozoa and more than 75% morphologically normal spermatozoa. Samples of 15 mL of each ejaculate were centrifuged twice at 1500× *g* for 10 min at room temperature (RT) (Rotofix 32A, Hettich Centrifuge UK, Newport Pagnell Buckinghamshire, UK) to collect seminal plasma. The resulting nine seminal plasma samples were randomly pooled three to three to generate three separate pools, each containing three seminal plasma samples. One tablet of a protease inhibitor cocktail (Roche protease inhibitor cocktail complete™, Basel, Switzerland) was added to each seminal plasma sample and the samples were then stored at 5 °C until the isolation of sEVs.

### 4.2. Isolation of sEVs

The isolation of sEVs was performed using the SEC-based protocol recently described by Martínez-Díaz et al. with slight modifications [16]. Briefly, the three seminal plasma samples (4 mL each) were centrifuged at 3200× *g* for 15 min at 4 °C (Sorvall™ BSTR40, Thermo Fisher Scientific, Waltham, MA, USA) to remove debris. The resulting supernatants were centrifuged at 20,000× *g* for 30 min at 4 °C (Sorvall™ Legend™ Micro 21R, Thermo Fisher Scientific). The resulting pellets and supernatants were processed separately for isolation of sEV subsets. Pellets were resuspended in 500 µL phosphate-buffered saline filtered at 0.22 µm (fPBS) and stored at 5 °C until SEC. Supernatants were diluted in fPBS (1:2, *v*:*v*), filtered (0.22 µm, Millex^®^ syringe filters) and reconcentrated by ultrafiltration (UF) using 3 kDa Amicon^®^ Ultra-4 mL centrifuge filters (UFC8003, Merck KGaA, Darmstadt, Germany) and multiple cycles of 3200× *g* centrifugation at 4 °C. Samples retained on the ultrafilter (≈2 mL) were stored at 5 °C until SEC.

SEC was performed using home-made columns consisting of 10 mL of Sepharose CL-2B^®^ (Sigma Aldrich^®^, Burlington, MA, USA, Merck KGaA) packed into 12 mL filtration tubes (Supelco^®^ Filtration Tubes 12 mL, Bellefonte, PA, USA). The supernatant and pellet samples from each 20,000× *g* centrifugation were separately subjected to SEC. Twenty eluted fractions of 500 µL each were collected sequentially in each SEC. The EV-enriched fractions (7 to 9) from each SEC were pooled to create a single sEV sample. Thus, two sEV samples were obtained from each of the three seminal plasma pools, one enriched in smaller sEVs (S-sEVs) from the SEC of the 20,000× *g* centrifugation supernatant and one enriched in larger sEVs (L-sEVs) from the SEC of the 20,000× *g* centrifugation pellet. In total, six samples of sEVs, three S-sEVs and three L-sEVs, were generated and stored at −80 °C (Ultra Low Freezer; Haier Inc., Qingdao, China) until used for either EV characterization or lipidomic analysis.

### 4.3. Characterization of sEVs

The sEVs from the three samples of S-sEVs and the three samples of L-sEVs were characterized according to the minimal information for studies of extracellular vesicles 2018 (MISEV2018) guidelines [17]. For this, orthogonal characterization including total protein concentration, particle concentration, particle size distribution, sEV morphology, sEV protein-specific markers, and sEV purity was performed as described below.

Total protein concentration was measured using the bicinchoninic acid assay (Thermo Scientific™ Pierce Micro BCA, Waltham, MA, USA) according to the manufacturer’s instructions. Each sEV sample was analyzed under two different conditions, lysed and unlysed. Lysis was performed by mixing (1:1, *v*:*v*) sEV samples with lysis solution (0.1% of Triton plus 0.1% of sodium dodecyl sulfate in fPBS) and the mixture was incubated at 37 °C for 30 min under gentle agitation (≈50 rpm). Absorbance was measured at a wavelength of 570 nm (PowerWave XS; BioTek Instruments, Winooski, VT, USA). Two technical replicates were analyzed for each of the six biological samples. Results are expressed as μg per mL.

Particle concentration was measured by NTA using a NanoSight LM10 (Malvern Instrument Ltd., Malvern, UK) equipped with a 405 nm laser and a complementary scientific semiconductor metal oxide camera. Measurements were analyzed using NTA software (version 3.3.; dev build 3.3.104) with min track length, max jump distance, defocus set to auto, and the detection threshold set to five. The camera level was set to 15 and five 30 second videos were recorded at a 30 fps. Two technical replicates were analyzed for each of the six biological samples.

Particle size distribution was measured by DLS analysis using a Zetasizer Nano ZS system (Malvern Panalytical, Malvern, UK) operating at 633 nm at 25 °C and recording the backscattered light at an angle of 173°. Samples of 50 µL were loaded into 10 mm cuvettes. Light scattering was recorded for 150 s and 3 measurements were taken per sample. Dispersion Technology v.5.10 software (Malvern Panalytical) was used to convert the DLS signal intensity to size distribution. Particle diameter was determined from the maximum peak of the normal function. The intensity-based distribution was recalculated to volume and the results recorded as volume and intensity size distributions. Three technical replicates were analyzed for each of the six biological samples.

The morphology of S-sEVs and L-sEVs was evaluated by TEM using a JEOL JEM 1011 microscope (JEOL Ltd., Tokyo, Japan). Sample preparation and visualization were performed according to the protocol described by Théry et al. with slight modifications [75]. Briefly, 10 µL of sample was fixed in 2% paraformaldehyde for 30 min and applied to carbon-coated copper grids for 15 min. Samples were washed with fPBS, fixed in 1% glutaraldehyde, and washed with distilled water. The samples were then counterstained with 1% uranyl acetate and immersed in 0.5% methylcellulose. After drying, the samples were imaged at 80 kV.

The expression of the specific protein markers CD63 and HSP90β and the purity of the sEV samples were analyzed by flow cytometry using a high-sensitive flow cytometer (CytoFLEX S, Beckman Coulter, Life Sciences Division Headquarters, Indianapolis, IN, USA) equipped with violet (405 nm), blue (488 nm), yellow (561 nm), and red (638 nm) lasers according to the technical procedure described by Barranco et al. [34]. The accuracy of the flow cytometer for EV input and counting was confirmed using recombinant EVs expressing green fluorescent protein on their membrane surface (SAE0193, Merck). The optical setup was adjusted to use the side scatter (SSC) information from the 405 nm laser (violet SSC-A). The forward scatter (FSC) and violet SSC-A were set to a logarithmic scale and the fluorescence channels were set to a logarithmic gain. Samples were analyzed in low flow mode (10 μL/min) with a minimum of 10^4^ events per sample. Distilled water was used as the sheath fluid, while 0.1 μm-fPBS was used to ensure removal of background noise. Analysis was adjusted for intact sEVs. For this, samples were incubated with CellTrace™ CFSE (C34554, Thermo Fisher Scientific) and only CFSE-positive particles were considered sEVs.

Flow cytometry analysis was performed according to the ISEV guidelines [76]. Enrichment of proteins belonging to the three categories established by MISEV2018 [17] was assessed. Specifically, CD63 (anti-CD63-FITC, clone REA1055, Miltenyi Biotec, Bergisch Gladbach, Germany) belonging to category 1; HSP90β (anti-HSP90β-PE, ADI-SPA-844PE-050, Enzo Life Sciences, Farmingdale, NY, USA) belonging to category 2; and albumin (anti-swine albumin-FITC, CLFAG16140, Cedarlane, Burlington, MA, USA) belonging to category 3, that of non-EV protein markers. 

All relevant data from the experiments were submitted to the EV-TRACK database (EV-TRACK ID: EV240049) [77].

### 4.4. Lipidomic Analysis

Lipidomic analyses were performed by the Research Unit on Bioactive Molecules (RUBAM) of the Institute of Advanced Chemistry of Catalonia (IQAC, Barcelona, Spain), member of the Spanish National Research Council. Lipid extraction and lipid identification and quantification were performed as described by Simbari et al., with minor modifications [78].

#### 4.4.1. Determination of Total Protein Concentration

Total protein concentration of S-sEVs and L-sEVs was determined by bicinchoninic acid assay (Thermo Scientific™ Pierce Micro BCA) using BSA as a standard according to the manufacturer’s instructions. Samples were then frozen, lyophilized, and resuspended in 0.2 mL of MilliQ water.

#### 4.4.2. Lipid Extraction

A total of 750 µL of a chloroform-methanol (2:1, *v*:*v*) solution containing internal standards (16:0 D31_18:1 phosphocholine, 16:0 D31_18:1 phosphoethanolamine, 16:0 D31-18:1 phosphoserine, 17:0 lyso-phosphocholine, 17:1 lyso-phosphoethanolamine, 17:1 lyso-phosphoserine, 17:0 D5_17:0 diacylglycerol, 17:0/17:0/17:0 triacylglycerol and C17:0 choresteryl ester, N-dodecanoylsphingosine, N-dodecanoylglucosylsphingosine, N-dodecanoylsphingosylphosphorylcholine, 0.2 nmol each, and 2 nmol stigmasterol, from Avanti Polar Lipids, Alabaster, AL, USA) were added to 0.2 mL of S-sEVs and L-sEVs.

Samples were vortexed and sonicated until they appeared dispersed and extracted at 48 °C overnight and cooled. Samples were then evaporated to dryness and stored at −80 °C until analysis. Prior to analysis, 150 µL of methanol was added to the samples, centrifuged at 13,000× *g* for 5 min and 130 µL of the supernatant was transferred to a new vial and injected.

#### 4.4.3. Liquid Chromatography–High-Resolution Mass Spectrometry

Lipids were analyzed by liquid chromatography high-resolution mass spectrometry (LC–HRMS). LC–HRMS analysis was performed using an Acquity ultra-high-performance liquid chromatography (UHPLC) system (Waters Co, Wexford, Ireland) coupled to a time-of-flight detector (LCT Premier XE). Full scan spectra were acquired from 50 to 1800 Da, and individual spectra were summed to produce data points every 0.2 s. Mass accuracy at a resolution of 10,000 and reproducibility were maintained using an independent reference spray (leucine enkephalin) via the LockSpray interference. Lipid extracts were injected onto an Acquity UHPLC BEH C8 column (1.7 µm particle size, 100 mm × 2.1 mm, Waters Co) at a flow rate of 0.3 mL/min and a column temperature of 30 °C. The mobile phases were methanol with 2 mM ammonium formate and 0.2% formic acid (A)/water with 2 mM ammonium formate and 0.2% formic acid (*v*:*v*) (B). A linear gradient was programmed as follows: 0.0 min: 20% B; 3 min: 10% B; 6 min: 10% B; 15 min: 1% B; 18 min: 1% B; 20 min: 20% B; 22 min: 20% B. For HRMS analysis, the capillary voltage was set to 3.0 kV, the desolvation temperature was set to 350 °C, and the desolvation gas flow was set to 600 L/h.

Positive identification of compounds was based on the accurate mass measurement with an error <5 ppm and their LC retention time, compared to that of a standard (92%). Quantification was performed on the extracted ion chromatogram of each compound, using 50 mDa windows. Linear dynamic range was determined by injecting mixtures of internal and natural standards as described above. In the absence of authentic standards, identification was made by accurate mass measurement, elemental composition, calculated error, double bond equivalents, and retention time. The relative amount of each lipid species was measured as pmol equivalents to available standards, normalized to protein content and expressed as pmol per mg protein. The LIPID-MAPS consortium lipid nomenclature is used in this paper [79,80].

### 4.5. Statistical Analysis

Statistical analysis was performed using GraphPad Prism version 10.1.0 for Windows (GraphPad Software, San Diego, CA, USA). The normal distribution of the data was assessed using the Shapiro–Wilks test. Data were analyzed by paired *t*-test or one-way ANOVA followed by Tukey’s multiple comparison test for comparisons between two or more groups, respectively. The results of the sEV characterization are presented as the mean ± SD, unless otherwise noted. Statistical differences were considered when *p* values were <0.05.

## 5. Conclusions

In conclusion, porcine seminal EVs have a complex lipid composition, including 157 lipid species belonging to 14 different lipid classes. Most of the identified lipids were structural to cell membranes. However, some diacylglycerol and triacylglycerol species were also identified, which may originate from circulating free lipoproteins in seminal plasma bound to the sEV membrane. The two subsets of sEVs of different sizes showed qualitative and quantitative differences in lipid profile. The most notable quantitative differences were in sphingomyelins and ceramides, with the former in higher proportions in small sEVs and the latter in large sEVs, differences that may reveal different biogenesis pathways.

## Data Availability

All relevant data from the experiments were submitted to the EV-TRACK database (EV-TRACK ID: EV240049). Lipid species identified and quantified in both sEV subsets were uploaded to the Vesiclepedia database (Study ID: 3594). Data are available at the Zenodo Repository (https://www.zenodo.org, accessed on 3 June 2024) and can be accessed directly via its project DOI (https://doi.org/10.5281/zenodo.11448921).

## Supplementary Materials

The following supporting information can be downloaded at: https://www.mdpi.com/article/10.3390/ijms25137492/s1.
